# *ReUseData*: an *R/Bioconductor* tool for reusable and reproducible genomic data management

**DOI:** 10.1186/s12859-023-05626-0

**Published:** 2024-01-03

**Authors:** Qian Liu, Qiang Hu, Song Liu, Alan Hutson, Martin Morgan

**Affiliations:** Department of Biostatistics and Bioinformatics, Roswell Park Comprehensive Cancer Center, Buffalo, NY 14263 USA

**Keywords:** Genomic data, Data reusability, Data reproducibility, Data management, Common Workflow Language

## Abstract

**Background:**

The increasing volume and complexity of genomic data pose significant challenges for effective data management and reuse. Public genomic data often undergo similar preprocessing across projects, leading to redundant or inconsistent datasets and inefficient use of computing resources. This is especially pertinent for bioinformaticians engaged in multiple projects. Tools have been created to address challenges in managing and accessing curated genomic datasets, however, the practical utility of such tools becomes especially beneficial for users who seek to work with specific types of data or are technically inclined toward a particular programming language. Currently, there exists a gap in the availability of an R-specific solution for efficient data management and versatile data reuse.

**Results:**

Here we present *ReUseData*, an *R* software tool that overcomes some of the limitations of existing solutions and provides a versatile and reproducible approach to effective data management within R. *ReUseData* facilitates the transformation of ad hoc scripts for data preprocessing into Common Workflow Language (CWL)-based data recipes, allowing for the reproducible generation of curated data files in their generic formats. The data recipes are standardized and self-contained, enabling them to be easily portable and reproducible across various computing platforms. *ReUseData* also streamlines the reuse of curated data files and their integration into downstream analysis tools and workflows with different frameworks.

**Conclusions:**

*ReUseData* provides a reliable and reproducible approach for genomic data management within the *R* environment to enhance the accessibility and reusability of genomic data. The package is available at *Bioconductor* (https://bioconductor.org/packages/ReUseData/) with additional information on the project website (https://rcwl.org/dataRecipes/).

**Supplementary Information:**

The online version contains supplementary material available at 10.1186/s12859-023-05626-0.

## Background

The growing volume and complexity of genomic data resources [1–3] present significant challenges for efficient data management and reuse, particularly with the widespread adoption of FAIR (findability, accessibility, interoperability, and reusability) [4] data principles and organizational requirements for Data Management and Sharing Plans [5–8]. Typically, public genomic data resources undergo similar preprocessing steps across various research projects. This often results in the creation of redundant, duplicate, or inconsistent datasets, leading to the inefficient use of computational resources due to the absence of standardized software tracking and data annotation strategies. Moreover, a substantial portion of data analysis tasks consist of crafting ad hoc data processing scripts. These scripts often lack reproducibility, which hampers both scientific rigor and research collaboration. These concerns are particularly pertinent for bioinformaticians, especially those operating within core facilities, due to their involvement in multiple data analysis projects using the same public data resource. Similar scenarios can also apply to researchers working in laboratory settings, where experiment data may be analyzed by different individuals using diverse approaches.

Many databases and associated tools have been developed to address common challenges in genomic data access and management. For instance, the Galaxy Data Manager framework [9, 10] effectively oversees reference data linked to specific Galaxy platform tools, which can also be accessed and utilized by other tools and platforms. Ensembl's core software and APIs [11, 12] provide access to stable sources of genomic sequence and annotation files. The SRA toolkit [13] takes care of sequence data storage, compression, and format conversion. Refgenie [14] is designed for the management and sharing of reference genomes and related files. The *R* tool *tximeta* [15] utilizes reference sequence checksums to identify provenance in RNA-seq data and ensure its computational reproducibility. These tools have offered valuable solutions that cater to users with specific interests in certain data types or particular programmatic preference. GoGetData [16] was developed to resolve issues related to standardized and reproducible access to a variety of genomic data. It offers a powerful and user-friendly command-line tool by leveraging the Conda and BioConda [17] ecosystem and integrating tailored enhancements to optimize efficient data handling and management. *AnnotationHub* [18] provides an easy-to-use *R* interface that helps users access and use curated genomic data from well-established repositories like UCSC and Ensembl. With efficient management of both local and global data, it streamlines data integration in the downstream analysis within the *R* environment. However, the data utilization is not optimized for integration within other platforms such as workflow-based analysis pipelines. We have recognized the need for an R-based solution that enables efficient data management and more flexible reuse across a variety of approaches and platforms.

In this context, we introduce *ReUseData*, an *R* [19] software tool designed to tackle some of the limitations while bridging the gap by providing a reproducible and flexible solution within the *R* environment. ReUseData provides an easy-to-use *R* approach for the management of all reusable data, including both laboratory-specific experiment data and the curation of publicly available genomic data resources. It also aims to facilitate efficient data reuse across various platforms, not confined within *R/Bioconductor*. *ReUseData* enables the transformation of ad hoc scripts for data preprocessing into Common Workflow Language (CWL)-based [20] data recipes, allowing for reproducible generation of curated data files in generic formats. It adds standardized entries as well as user-defined attributes to ensure comprehensive data provenance and user-friendly data discovery. Moreover, *ReUseData* incorporates functions aimed at fostering the reuse of these data files. For instance, it permits the labeling of different curated datasets with shared software identifiers and their aggregation into formats conducive for integration with downstream analytical tools, including *R*/*Bioconductor* [21, 22] packages, command-line utilities, and analysis workflows supported by various frameworks such as CWL, Workflow Description Language (WDL) [23], Nextflow [24] or snakemake [25].

## Implementation

In previous work, we have developed two packages: *Rcwl* and *RcwlPipelines* [26], with the goal of constructing a *Bioconductor* toolchain for reproducible bioinformatics pipelines. By transforming conventional command-line software tools into modularized *R* tool recipes anchored in the CWL framework, these packages empower users to effortlessly implement reproducible data processing workflows directly inside the *R* environment. Expanding upon this groundwork, we have taken the concept of tool recipes from *RcwlPipelines* and evolved them into data recipes, with a focus on streamlining the preprocessing of reusable genomic data resources. One distinctive feature of *ReUseData* is its ability to handle multiple software tools within a single recipe, which aligns with common scenario in data processing scripts, while CWL and Rcwl features modularized tool recipes where each tool is integrated individually through docker or Conda, and then connected to form a pipeline.

Inherited from *Rcwl*, the data recipes generated from *ReUseData* are inherently self-contained and independent of pre-existing software installations, ensuring seamless portability and reproducibility across various computing platforms equipped with *R*. When working on a system without the necessary data preprocessing tools, the recipe evaluation function (*getData*) internally initiates a reproducible Conda environment [27] where the CWL runner and required software tools sourced from popular Docker registries, e.g., SAMtools [28], STAR [29] (gene expression summary), and GATK [23] (variant calling), are installed and run. it's important to emphasize that all of these Conda-related operations occur seamlessly within the *R* functions, requiring no action or prior knowledge from users to fully utilize this feature.

An important innovation brought by *ReUseData* is its standardized approach to managing meta information, encompassing data origin, software tool version, and secondary data files associated with the primary data. The evaluation of data recipes generates curated data files at a user-designated file path, complemented by annotation files housing metadata for preserving data provenance (Fig. 2B). The metadata includes standardized data entries, such as data origin, software version, file path and date, along with user-defined attributes like keywords and software tags. These elements collectively expedite effective data exploration, retrieval, and sharing.

*ReUseData* introduces distinctive functionalities for handling curated data files derived from data recipes, encouraging their reuse across various projects. The curated data can be represented as an *R* object containing essential information such as the file paths etc. Utility functions (*dataTag*, *toList*) were developed to tag a specific group of dataset, e.g., with a software name which takes the dataset as input, and prepares them into appropriate format for streamlined integration into downstream analysis tools, such as *R/Bioconductor* packages, analysis workflows within *R* enabled by *Rcwl/RcwlPipelines* suite of tools and pipelines, or directly into workflow frameworks such as CWL and WDL, that are available in local computer, HPC or cloud computing platforms. *ReUseData* offers a Google Bucket (https://storage.cloud.google.com/reusedata) for archiving the commonly used curated data resources, which can be downloaded programmatically from your local computer, or used directly on cloud computing platforms with minimum cloud-to-cloud latency, further advancing the goals of data sharing and reuse.

## Results

### Access, curation and management of genomic data using *ReUseData*

**Fig. 1:**
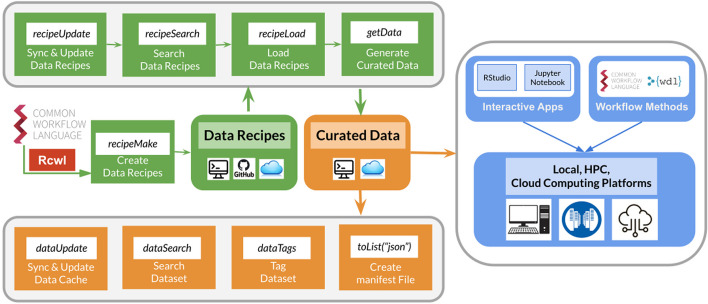
Overview of the *ReUseData* functionalities, deliverables, and their usage in local or cloud-based data analysis tasks. Two main sets of functions are present for the management of data recipes (in green) and curated data files (in orange). Self-contained data recipes are highly portable and reproducible across diverse computing platforms (local computer, HPC, cloud computing platforms). Curated datasets that were generated from *ReUseData* recipes can be smoothly integrated into downstream data analysis that were based on command-line tools, *R/Bioconductor* packages, and workflow methods

*ReUseData* consists of two major categories of functions for managing data recipes and curated data resources (Fig. 1). It uses a caching system with a database infrastructure through *BiocFileCache* [30] to manage existing data recipes, enabling easy updating, searching, and loading within *R*. A separate caching system is used to manage and index the curated data files (in their generic format) that can be locally generated or found on the cloud.

**Fig. 2:**
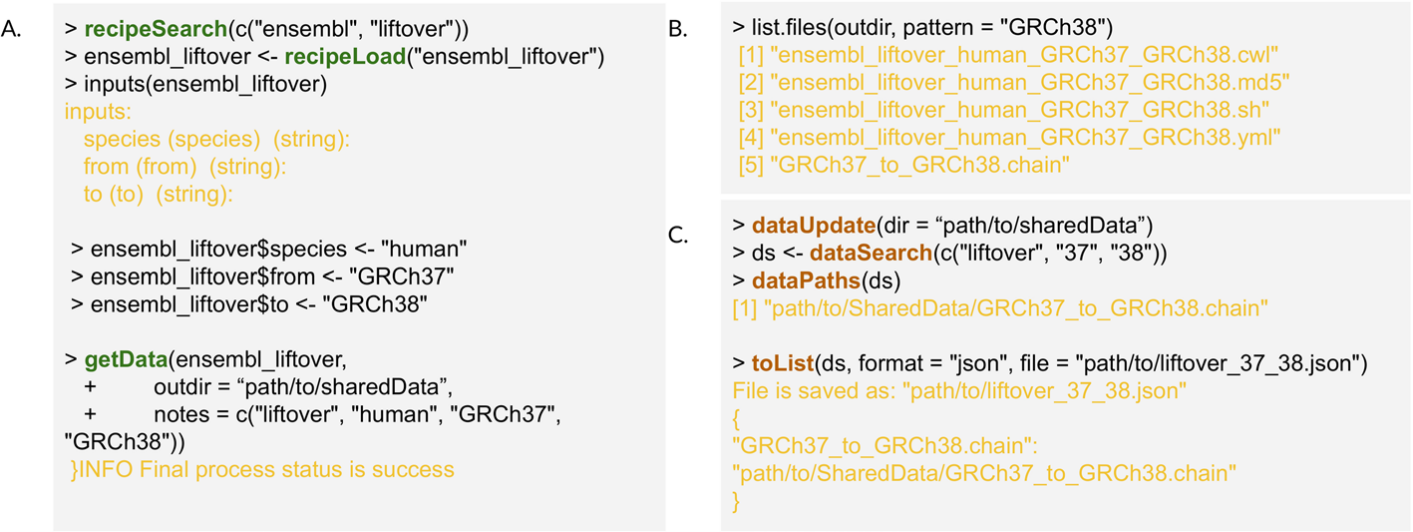
Using the *ReUseData* data recipes to reproducibly generate and efficiently manage curated genomic datasets

*ReUseData* provides *R* functionalities to help standardize the access, curation and management of genomic datasets and facilitate data reproducibility and reusability in different projects and platforms. Development and use of *ReUseData* involves four steps: (i) (developers only) write or prepare an existing data processing script and convert it into a data recipe by specifying input parameters, output extraction patterns and software tools required for data processing (*recipeMake*). (ii) Synchronize and search existing data recipes for data downloading/curation with multiple keywords, and load into *R (recipeSearch, recipeLoad)*. (iii) Check and assign values for required input parameters, and then evaluate the recipe to generate curated data sets with standardized annotations (*getData*). (iv) Search data with multiple keywords, and pass data directly into *R/Bioconductor* packages, specific software tools (e.g., GATK) or data analysis workflows (e.g., based on CWL or WDL) (*dataUpdate*, *dataSearch*, *getCloudData*, *toList*). Additional utility functions assist users in tailoring data recipes and extracting specific information from cached data (Table 1). Figure 2 shows the pseudo code using the data recipe for downloading and unzipping genome liftover files from Ensembl. This process shows how *ReUseData* functionalities are employed to generate, manage, and utilize curated genomic files. Expanded examples for demonstration of package use and other resources are provided in the additional file [see Additional file 1: ReUseData_supplement.pdf].

**Table 1:** A catalog of *ReUseData* functions

Category	Function	Description
data recipe	recipeMake	Create data recipe from scratch or existing shell script
	recipeUpdate	Synchronize and update the data recipes GitHub repository or user-specified private GitHub repository
	recipeSearch	Search existing data recipes in the cache path of "recipeUpdate"
	recipeLoad	Load one or more data recipes into the *R* environment
	recipeNames	S4 method for "recipeHub" object (data recipe representation in R). Returns the recipe name(s)
	getData	Evaluation of data recipe to generate curate data file(s) of interest
Curated data	dataUpdate	Function to update the local data records by reading the yaml files in the specified directory recursively
	dataSearch	Search specific data files in the local data caching system using one or more keywords
	dataNames	S4 method for "dataHub" object (data files representation in R). Returns the name(s) of data files
	dataNotes	S4 method for "dataHub" object. Returns the "notes" of the data that was defined in "getData"
	dataParams	S4 method for "dataHub" object. Returns the input parameter values for the corresponding data recipe
	dataPaths	S4 method for "dataHub" object. Returns the file path(s) of corresponding data files
	dataTags	S4 method for "dataHub" object. Returns the tags of data files (if exist)
	dataTags < -	Function to assign a string tag to specific data files
	dataYml	S4 method for "dataHub" object. Returns the yaml file path(s) for corresponding data file(s)
	getCloudData	Download the pre-generated curated data sets from *ReUseData* Google Cloud Bucket

(A) Pre-built public data recipes in *ReUseData* or user-defined private data recipes in a GitHub repository are easily retrieved with `recipeSearch` and `recipeLoad` functions. Once we assign value for the input parameters, the data recipe is ready to be evaluated to reproducibly generate the needed data file(s) on any computing platform equipped with *R*. Arbitrary “notes” can be added to facilitate subsequent data retrieval. (B) The recipe evaluation generates the needed data files, as well as meta files to help preserve data provenance. “.yml” file records both standardized data annotations, such as data origin, software version, output file path and date, and user-defined attributes, such as keywords and software tags. All the information helps track the data resources, processing tools etc. for reproducible data generation and efficient data retrieval. “.sh” file records the low-level shell script for data processing. “.cwl” file is the cwl wrapup of the shell script. “.md5” checksum file is to check and verify the integrity of generated data files. (C) The data files that were generated using *ReUseData* functions are cached in a user-specified shared folder for your working group, and can be easily retrieved using keywords (from recipe name, data name, user-added notes, etc.). Data can also be prepared with standardized formats (e.g., json, yaml) for data analysis workflows. Recipe related functions are in green; Data managing functions are in Orange. *R* outputs chunks are in light yellow.

### Pre-built data recipes

We have pre-built data recipes using *ReUseData* for common preprocessing of genomic data resources. The different types of data recipes offer simple customization, serving as templates for users to develop their customized data recipes under different situations.

The most common and suggested type is a data recipe with input parameters (species, versions, etc.) to accommodate multiple data sets from the same/similar sources. For example, as an initial step of bulk or single-cell RNASeq analysis, the “gencode_transcripts” recipe downloads from GENCODE, unzips and indexes the transcript fasta file for species of human or mouse with different versions. A simple data recipe without any input parameters can be written to manage the processing of a specific file from a fixed data source, For example, the “gencode_genome_grch38” recipe downloads the human genome file “GRCh38.primary_assembly.genome.fa.gz” from GENCODE release 42. For more complex data processing where multiple software tools are involved, or secondary files are to be generated and collected, the raw way of building a data recipe using *Rcwl* functions gives more flexibility and power. The “reference_genome” example recipe demonstrates the process of downloading, formatting, and indexing reference genomes using samtools, picard, and bwa, and manages multiple secondary files besides the main fasta file for subsequent data use. These pre-built data recipes (Table 2) can be easily queried and evaluated in *R* for direct use.

**Table 2:** Catalog of pre-built data recipes in *ReUseData*

*ReUseData* recipes	Description
bowtie2_index	Use "bowtie2-build" to index a specific reference genome
echo_out	Print a character string and output as a txt file
ensembl_liftover	Download and unzip genome liftover file from Ensembl
gcp_broad_gatk_hg19	Download file in GATK annotation bundle for hg19 from GCP
gcp_broad_gatk_hg38	Download file in GATK annotation bundle for hg38 from GCP
gcp_gatk_mutect2_b37	Download file in GATK mutect2 bundle for b37 from GCP
gcp_gatk_mutect2_hg38	Download file in GATK mutect2 bundle for hg38 from GCP
gencode_annotation	Download and unzip annotation files from gencode
gencode_genome_grch38	Download human genome GRCh38 from GENCODE release 42
gencode_transcripts	Download, unzip, and index transcripts files from gencode
hisat2_index	Use "hisat2-build" to build the index files
reference_genome	Download, format and index reference genome file with samtools, picard and bwa
salmon_index	Use "Salmon index" command to build salmon index for your transcriptome
STAR_index	Use STAR command to build genome index for STAR alignment
ucsc_database	Download genome annotation file from UCSC database using the golden path

With the purpose of providing a template repository, we plan to add more pre-built recipes when different situations arise. *ReUseData* provides functions to help users develop their own set of data recipes. These can be deposited in a private GitHub repository, accessible exclusively to a specific workgroup, or contributed back to *ReUseData* for broader accessibility, benefiting researchers in similar domains. To be included in *ReUseData*, additional meta information will be required for each data recipe, such as the links to data origin and source code, description of valid parameter values and demonstrative code of using, so that a landing page of the data recipe can be created on the *ReUseData* portal. This can be facilitated by the *RcwlMeta* package that is available on our GitHub organization.

### Cloud-sharing of curated genomic data

By evaluating the pre-built data recipes, we have successfully produced curated data files that correspond to specific data sources, species, and versions. These files have been stored in a Google Cloud Bucket (https://storage.cloud.google.com/reusedata). Users have the opportunity to contribute their curated genomic data files, subject to evaluation and validation, if they are associated with a shared data recipe via *ReUseData*. The datasets are now organized by data recipes, each having distinct entries for input parameters. Users can access these files programmatically through *ReUseData* or directly on cloud computing platforms like Terra [23, 31] and CGC [32]. These platforms offer minimal latency for cloud-to-cloud data transfers, enhancing the overall accessibility and usability of the data.

### Project website and documentations

We have developed the website (https://rcwl.org/) as a central hub for *ReUseData* and *Rcwl/RcwlPipelines* resources. It encompasses dedicated sections for *ReUseData* data recipes and *Rcwl* pipelines, where distinct landing pages are provided for users to explore the specifications and instructions of each recipe.

While *ReUseData* data recipes inherently encompass data processing pipelines involving multiple tools, the recipe landing pages feature diagrams illustrating the flow from input parameters to processing tools to output data files. These landing pages also include explanations of input and output parameters, and verified code snippets for step-by-step guidance in utilizing the data recipe or downloading pre-built curated data from the Google Cloud bucket. Additionally, the pages offer links to recipe source code and original data sources for licensing and registration purposes wherever possible.

The website provides tutorial e-books and case studies that show how to use CWL pipelines in* R*. Leveraging the same CWL-based architecture, the reproducible genomic data files generated from *ReUseData* data recipes can seamlessly integrate with tool and pipeline recipes in *RcwlPipelines*, forming a cohesive framework for reproducible data management and analysis within the unified *R* working environment.

We have also created supplementary resources to guide users through *ReUseData* and the *Rcwl* ecosystem, which include package vignettes, workshop materials available on GitHub, and linked videos on YouTube.

## Conclusion

We have introduced *ReUseData*, an *R/Bioconductor* package designed to offer a reliable and reproducible approach for accessing and managing genomic data within the user-friendly *R* programming environment. Utilizing workflow and recipe methodologies in conjunction with established initiatives like *Rcwl/RcwlPipelines* and leveraging the capabilities of the Conda environment, *ReUseData* also introduces innovative strategies to ensure data provenance and facilitate local/cloud data sharing, aiming to enhance the findability, accessibility, interoperability, and reusability of genomic data resources. The package is thoroughly documented, offering instructions and resources through *Bioconductor* vignettes, the project website, and cloud data sharing, while continuous improvements and expansions are planned based on community needs and requests.

### Supplementary Information

**Additional file 1. **ReUseData package resources and practical use.

## Data Availability

Project name: ReUseData Project home page: https://rcwl.org/dataRecipes/ (*ReUseData* website); https://bioconductor.org/packages/ReUseData (*Bioconductor*); https://storage.cloud.google.com/reusedata (Cloud Data). Operating system(s): Linux, macOS. Programming language: R Other requirements: None. License: GPL-3 Any restrictions to use by non-academics: None.
