# Supplementary material for: ReUseData: an R/Bioconductor tool for reusable and reproducible genomic data management
Source: BMC Bioinformatics. 2024 Jan 3;25:8. doi: 10.1186/s12859-023-05626-0 (PMC10765726; doi:10.1186/s12859-023-05626-0)
Supplement: Supplementary file 1 — Additional file 1. ReUseData package resources and practical use. [file 12859_2023_5626_MOESM1_ESM.pdf]

ReUseData: An R/Bioconductor Tool For Reusable and Reproducible Genomic Data Management

Qian Liu [1]***, Qiang Hu*** [1]***, Song Liu*** [1]***, Alan Hutson*** [1]***, and Martin Morgan*** [1]

[1]Roswell Park Comprehensive Cancer Center

2023-11-10

Contents

1 Project resources . . . 2
- 1.1 `ReUseData` portal . . . 2
- 1.2 Pre-built data recipes . . . 2
- 1.3 Cloud sharing of curated data . . . 2
- 1.4 Package installation . . . 3

2 Core functions for data recipes . . . 3
- 2.1 Recipe construction and evaluation . . . 3
- 2.2 Recipe caching and updating . . . 5
- 2.3 Recipe searching and loading . . . 6

3 Core functions for curated data . . . 8
- 3.1 Data generation . . . 8
- 3.2 Data caching, updating, and searching . . . 9
- 3.3 Data on the cloud . . . 11
- 3.4 Know your data . . . 12

4 SessionInfo . . . 13

In this supplement, we will demonstrate the use of the `ReUseData` functions in managing the data recipes and curated data resources. There are three major sections: 1) Project resources. 2) The package core functions for data recipes. 3) The package core functions for curated data management.

1 Project resources

1.1 `ReUseData` portal

The project website https://rcwl.org/dataRecipes/ provides a central hub for all the pre-built data recipes for data curation (downloading, unzipping, indexing, etc.) of commonly used public data resources. The website contains a search bar with autocompletion for convenient recipe searching. Each data recipe comes with a landing page, where there is the recipe description, the link to recipe source code, original data sources, annotation for the input and output parameters, and user instructions with example code chunks.

While these pre-built data recipes are ready for direct use, they also serve as templates for users to create their own recipes, say for protected data sets. Before installing `ReUseData`, we recommend that potential users browse this portal to get some idea about what it is and how it works.

If anyone is interested in the CWL workflow infrastructure of `ReUseData` in *R*, or running CWL pipelines in *R*, there are resources available on this main website https://rcwl.org/, such as the `Rcwl` tutorial e-book, more than 200 pre-built Rcwl tools and pipelines, and case studies of using `RcwlPipelines` in preprocessing single-cell RNA-seq data, etc.

1.2 Pre-built data recipes

The pre-built `ReUseData` recipe scripts are included in the package and also physically residing in a dedicated GitHub repository, which demonstrates the recipe construction in different situations. The most common case is that a data recipe can manage multiple data sets from the same/similar sources using input parameters (species, versions, etc.). For example, the `gencode_transcripts` recipe downloads from GENCODE, unzips and indexes the transcript fasta file for human or mouse with different versions. A simple data downloading (using `wget`) for a specific file can be written as a data recipe without any input parameters. For example, the data recipe `gencode_genome_grch38.R` downloads the human genome file `GRCh38.primary_assembly.genome.fa.gz` from GENCODE release 42.

If the data curation gets more complicated, say, multiple command-line tools are to be involved, and `conda` is to be used to install required packages, or some secondary files are to be generated and collected, the raw way of building a `ReUseData` recipe using `Rcwl` functions is recommended. It gives more flexibility and power to accommodate different situations. An example recipe is the `reference_genome`, which downloads, formats, and indexes reference genome data using tools of `samtools`, `picard`, and `bwa`, and manages multiple secondary files besides the main fasta file for later reuse.

1.3 Cloud sharing of curated data

With the pre-built data recipes for curation of commonly used public data resources, we have generated some curated data sets to share on the Google Cloud (https://storage.cloud.google.com/reusedata). These data sets can be used directly on the cloud computing platforms (e.g.,

Terra, CGC), which may benefit from the low latency of cloud-to-cloud data transfer. They can also be downloaded and added to your local data cache. The concomitant annotation files will also be downloaded automatically for subsequent data reuse.

Any recipe with cloud data available has on its landing page the example code chunk showing how to download the data (without evaluating the recipe yourself) and add them into the local data cache.

1.4 Package installation

1. Install the package from Bioconductor or GitHub.

```
if (!requireNamespace("BiocManager", quietly = TRUE))
    install.packages("BiocManager")
BiocManager::install("ReUseData")
## BiocManager::install("rworkflow/ReUseData")
```

```
library(ReUseData)
```

2. Install and load other necessary packages (for this demo) into the R session.

```
## BiocManager::install("Rcwl")
library(Rcwl)
```

2 Core functions for data recipes

Here we show the usage of 4 core functions `recipeMake`, `recipeUpdate`, `recipeSearch`, `recipeLoad` for constructing, updating, searching and loading of `ReUseData` recipes in *R*.

2.1 Recipe construction and evaluation

One can construct a data recipe from scratch or convert existing shell scripts for data processing into data recipes, by specifying input parameters and output globbing patterns using the `recipeMake` function. Then, the data recipe is represented in *R* as an S4 class `cwlProcess`. Upon assigning values to the input parameters, the recipe is ready to be evaluated to generate data of interest. Here we show two examples.

NOTE that software tool versions can be specified when creating a data recipe using the `requireTools` argument in `recipeMake`. For example, `recipeMake(..., requireTools = "samtools=1.3")`.

- Write a data recipe froms scratch

```
script <- '
  input=$1
  outfile=$2
  echo "Print the input: $input" > $outfile.txt
  '
rcp <- recipeMake(shscript = script,
                  paramID = c("input", "outfile"),
                  paramType = c("string", "string"),
                  outputID = "echoout",
```

```
                  outputGlob = "*.txt")
inputs(rcp)
#> inputs:
#>   input (string):
#>   outfile (string):
outputs(rcp)
#> outputs:
#> echoout:
#>   type: File[]
#>   outputBinding:
#>     glob: '*.txt'
```

```
rcp$input <- "Hello World!"
rcp$outfile <- "outfile"
outdir <- file.path(tempdir(), "SharedData")
res <- getData(rcp,
               outdir = outdir,
               notes = c("echo", "txt", "test"),
               prefix = "echo_hello_world", ## for concomitant annotation files
               showLog = FALSE)
```

Let's take a look at the output file, which were successfully generated in the user-specified directory and grabbed through the `outputGlob` argument.

```
res$out
#> [1] "/tmp/Rtmp6l0Pi8/SharedData/outfile.txt"
readLines(res$out)
#> [1] "Print the input: Hello World!"
```

- Convert an existing shell script into a data recipe

The script can be shell or other ad hoc data processing scripts. Here we are using the shell script that download and index transcript annotation files from GENCODE for bulk or single-cell RNAseq analysis.

```
shfile <- system.file("extdata", "gencode_transcripts.sh", package = "ReUseData")
readLines(shfile)
#>  [1] "species=$1"
#>  [2] "version=$2"
#>  [3] "if [ $species == 'human' ] && [ $version -gt 22 ]"
#>  [4] "then"
#>  [5] "  trans='transcripts'"
#>  [6] "else "
#>  [7] "  trans='pc_transcripts'"
#>  [8] "fi"
#>  [9] "wget https://ftp.ebi.ac.uk/pub/databases/gencode/Gencode_$species/release_$version/gencode.v$versio
#> [10] "gzip -d gencode.v$version.$trans.fa.gz"
#> [11] "samtools faidx gencode.v$version.$trans.fa"
rcp <- recipeMake(shscript = shfile,
                  paramID = c("species", "version"),
                  paramType = c("string", "string"),
                  outputID = "transcripts",
```

```
                    outputGlob = "*transcripts.fa*"
                    )
```

Users can then assign values to the input parameters, and evaluate the recipe (`getData`) to generate data of interest.

```
inputs(rcp)
#> inputs:
#>   species (string):
#>   version (string):
rcp$species <- "mouse"
rcp$version <- "M31"
res <- getData(rcp,
        outdir = outdir,
        notes = c("gencode", "transcript", "mouse", "M31"))
```

The file path to the newly generated data set can be easily retrieved.

```
res$output
#> [1] "/tmp/Rtmp6l0Pi8/SharedData/gencode.vM31.pc_transcripts.fa"
#> [2] "/tmp/Rtmp6l0Pi8/SharedData/gencode.vM31.pc_transcripts.fa.fai"
```

The user-created data recipes can be then deposited on a private GitHub repository, exclusively accessible by a specific workgroup, or contributed back to `ReUseData` for broader accessibility to benefit researchers in similar research domains. In this case, additional meta information will be required for each data recipe, such as the links to data origin and source code, description of valid parameter values and demonstrative code of using, so that a landing page of the data recipe can be created on the ReUseData portal. This can be facilitated by the `RcwlMeta` package by communicating with the developer team.

2.2 Recipe caching and updating

`recipeUpdate()` creates a local cache (if first time use) for data recipes that are in specified GitHub repository, and it syncs and updates data recipes from the GitHub repo to the local caching system, so any newly added recipes can be readily accessed and loaded into *R*.

NOTE:

- The `cachePath` argument needs to match within `recipeUpdate`, `recipeLoad`, and `recipeSearch` functions.
- Use `force=TRUE` when any previously cached recipes are updated.

```
## First time use
recipeUpdate(cachePath = "ReUseDataRecipe")
#> recipeHub with 17 records
#> cache path:  /home/qian/.cache/R/ReUseDataRecipe
#> # recipeSearch() to query specific recipes using multipe keywords
#> # recipeUpdate() to update the local recipe cache
#>
#>             name
#>   BFC1906 | bowtie2_index
#>   BFC1907 | echo_out
```

```
#>   BFC1908 | ensembl_liftover
#>   BFC1909 | gcp_broad_gatk_hg19
#>   BFC1910 | gcp_broad_gatk_hg38
#>   ...       ...
#>   BFC1918 | reference_genome
#>   BFC1919 | salmon_index
#>   BFC1920 | STAR_index
#>   BFC1921 | ucsc_database
#>   BFC1922 | ReUseDataRecipe-master
```

`recipeUpdate` returns a `recipeHub` object with a list of all available recipes. One can subset the list with `[` and use getter functions `recipeNames()` to get the recipe names, which can then be passed to the `recipeSearch()` or `recipeLoad()`.

```
rh <- recipeUpdate()
is(rh)
#> [1] "recipeHub"             "cwlHub"                "BiocFileCacheReadOnly"
#> [4] "BiocFileCacheBase"
rh[1]
#> recipeHub with 1 records
#> cache path:  /home/qian/.cache/R/ReUseDataRecipe
#> # recipeSearch() to query specific recipes using multipe keywords
#> # recipeUpdate() to update the local recipe cache
#>
#>             name
#>   BFC1906 | bowtie2_index
recipeNames(rh)
#>  [1] "bowtie2_index"          "echo_out"               "ensembl_liftover"
#>  [4] "gcp_broad_gatk_hg19"    "gcp_broad_gatk_hg38"    "gcp_gatk_mutect2_b37"
#>  [7] "gcp_gatk_mutect2_hg38"  "gencode_annotation"     "gencode_genome_grch38"
#> [10] "gencode_transcripts"    "hisat2_index"           "meta_gcp.csv"
#> [13] "reference_genome"       "salmon_index"           "STAR_index"
#> [16] "ucsc_database"          "ReUseDataRecipe-master"
```

2.3 Recipe searching and loading

Cached data recipes can be searched using multiple keywords to match the recipe name. It returns a `recipeHub` object with a list of recipes matching.

```
recipeSearch("gencode")
#> recipeHub with 3 records
#> cache path:  /home/qian/.cache/R/ReUseDataRecipe
#> # recipeSearch() to query specific recipes using multipe keywords
#> # recipeUpdate() to update the local recipe cache
#>
#>             name
#>   BFC1913 | gencode_annotation
#>   BFC1914 | gencode_genome_grch38
#>   BFC1915 | gencode_transcripts
recipeSearch(c("STAR", "index"))
#> recipeHub with 1 records
```

```
#> cache path:  /home/qian/.cache/R/ReUseDataRecipe
#> # recipeSearch() to query specific recipes using multipe keywords
#> # recipeUpdate() to update the local recipe cache
#>
#>              name
#>   BFC1920 | STAR_index
```

Recipes can be directly loaded into *R* using the `recipeLoad` function with a user assigned name (or the original recipe name, see below for details). Once the recipe is successfully loaded, a message will be returned with a link to the recipe landing page on `ReUseData` portal with detailed user instructions. **Make sure to check the instructions on eligible input parameter values before recipe evaluation.**

```
rcp <- recipeLoad("STAR_index")
#> Note: you need to assign a name for the recipe: rcpName <- recipeLoad('xx')
#> Data recipe loaded!
#> Use inputs() to check required input parameters before evaluation.
#> Check here: https://rcwl.org/dataRecipes/STAR_index.html
#> for user instructions (e.g., eligible input values, data source, etc.)
```

NOTE Use `return=FALSE` if you want to keep the original recipe name, or if multiple recipes are to be loaded.

```
recipeLoad("STAR_index", return = FALSE)
```

```
identical(rcp, STAR_index)
#> [1] TRUE
```

```
recipeLoad(c("STAR_index", "gencode_annotation"), return=FALSE)
#> Data recipe loaded!
#> Use inputs(STAR_index) to check required input parameters before evaluation.
#> Check here: https://rcwl.org/dataRecipes/STAR_index.html
#> for user instructions (e.g., eligible input values, data source, etc.)
#> Data recipe loaded!
#> Use inputs(gencode_annotation) to check required input parameters before evaluation.
#> Check here: https://rcwl.org/dataRecipes/gencode_annotation.html
#> for user instructions (e.g., eligible input values, data source, etc.)
```

It's important to check the required `inputs()` of the recipe and the recipe landing page for eligible input parameter values before evaluting the recipe to generate data of interest.

```
inputs(STAR_index)
#> inputs:
#>   ref (reference genome)   ( string|File ):
#>   gtf (GTF)   ( string|File ):
#>   genomeDir (genomeDir)  (string):
#>   threads (threads)  (int):
#>   sjdb (sjdbOverhang)  (int):  100
inputs(gencode_annotation)
#> inputs:
#>   species (species)  (string):
#>   version (version)  (string):
```

3 Core functions for curated data

Here we introduce the core functions of `ReUseData` for data management and reuse: `getData` (or `getCloudData`) for reproducible data generation (or downloading from Google bucket), `dataUpdate` for syncing and updating the data cache, and `dataSearch` for multi-keywords searching of a data set of interest.

3.1 Data generation

Once we have a data recipe, we will first need to check the landing page for the recipe annotation, e.g., eligible values for each input parameter.

```
ensembl_liftover <- recipeLoad("ensembl_liftover")
inputs(ensembl_liftover)
#> inputs:
#>   species (species)  (string):
#>   from (from)  (string):
#>   to (to)  (string):
```

Users can then assign values to the input parameters, and evaluate the recipe (`getData`) to generate data of interest. Users need to specify an output directory for all files (desired data files, and concomitant annotation files that are internally generated for data reuse). We encourage detailed notes for the data to be generated, which will be used for keywords matching in later data searches.

```
ensembl_liftover$species <- "human"
ensembl_liftover$from <- "GRCh37"
ensembl_liftover$to <- "GRCh38"

res <- getData(ensembl_liftover,
        outdir = outdir,
        notes = c("liftover", "human", "GRCh37", "GRCh38"))
```

The file path to the newly generated data set can be easily retrieved. It can also be retrieved using `dataSearch()` functions with multiple keywords once `dataUpdate()` is done.

```
res$output
#> [1] "/tmp/Rtmp6l0Pi8/SharedData/GRCh37_to_GRCh38.chain"
```

There are some automatically generated annotation files to help track the data recipe evaluation, including the `*.sh` to record the original shell script, `*.cwl` file as the official workflow script, which was internally submitted for data recipe evaluation, `*.yml` file as part of the CWL workflow evalution, which also records data annotations, and `*.md5` checksum file to check and verify the integrity of generated data files.

```
list.files(outdir, pattern = "GRCh38")
#> [1] "ensembl_liftover_human_GRCh37_GRCh38.cwl"
#> [2] "ensembl_liftover_human_GRCh37_GRCh38.md5"
#> [3] "ensembl_liftover_human_GRCh37_GRCh38.sh"
```

```
#> [4] "ensembl_liftover_human_GRCh37_GRCh38.yml"
#> [5] "GRCh37_to_GRCh38.chain"
```

The `*.yml` file contains information about recipe input parameters, the file path to the output file, the notes for the data set, and date and time for data generation. A later data search using `dataSearch()` will refer to this file for keywords matching.

```
readLines(res$yml)
#> [1] "species: human"
#> [2] "from: GRCh37"
#> [3] "to: GRCh38"
#> [4] "# output: /tmp/Rtmp6l0Pi8/SharedData/GRCh37_to_GRCh38.chain"
#> [5] "# notes: liftover human GRCh37 GRCh38"
#> [6] "# date: 2023-11-10 21:06:44"
```

3.2 Data caching, updating, and searching

`dataUpdate()` creates (if first itme use), syncs and updates the local cache for curated data sets. It finds and reads all the `.yml` files recursivelly in the provided data folder, creates a cache record for each data set that is associated (including newly generated ones with `getData()`), and updates the local cache for later data searching and reuse.

IMPORTANT: It is recommended that users create a specified folder for data archival (e.g., `file/path/to/SharedData`) that other group members have access to, and use sub-folders for different kinds of data sets (e.g., those generated from the same recipe).

```
(dh <- dataUpdate(dir = outdir, keepTags = FALSE))
#> dataHub with 4 records
#> cache path:  /home/qian/.cache/R/ReUseData
#> # dataUpdate() to update the local data cache
#> # dataSearch() to query a specific dataset
#> # Additional information can be retrieved using:
#> # dataNames(), dataParams(), dataNotes(), dataPaths(), dataTag() or mcols()
#>
#>              name
#>   BFC32852 | outfile.txt
#>   BFC32853 | GRCh37_to_GRCh38.chain
#>   BFC32854 | gencode.vM31.pc_transcripts.fa
#>   BFC32855 | gencode.vM31.pc_transcripts.fa.fai
#>             Path
#>   BFC32852 /tmp/Rtmp6l0Pi8/SharedData/outfile.txt
#>   BFC32853 /tmp/Rtmp6l0Pi8/SharedData/GRCh37_to_GRCh38.chain
#>   BFC32854 /tmp/Rtmp6l0Pi8/SharedData/gencode.vM31.pc_transcripts.fa
#>   BFC32855 /tmp/Rtmp6l0Pi8/SharedData/gencode.vM31.pc_transcripts.fa.fai
```

`dataUpdate` and `dataSearch` return a `dataHub` object with a list of all available or matching data sets. One can subset the list with `[` and use getter functions to retrieve the annotation information about the data, e.g., data names, parameters values to the recipe, notes, tags, and the corresponding yaml file.

```
dh[1]
#> dataHub with 1 records
#> cache path:  /home/qian/.cache/R/ReUseData
#> # dataUpdate() to update the local data cache
#> # dataSearch() to query a specific dataset
#> # Additional information can be retrieved using:
#> # dataNames(), dataParams(), dataNotes(), dataPaths(), dataTag() or mcols()
#>
#>                name        Path
#>   BFC32852 | outfile.txt /tmp/Rtmp6l0Pi8/SharedData/outfile.txt
## dh["BFC1"]  ## Use the "BFC" index for direct matching
dh[dataNames(dh) == "GRCh37_to_GRCh38.chain"]
#> dataHub with 1 records
#> cache path:  /home/qian/.cache/R/ReUseData
#> # dataUpdate() to update the local data cache
#> # dataSearch() to query a specific dataset
#> # Additional information can be retrieved using:
#> # dataNames(), dataParams(), dataNotes(), dataPaths(), dataTag() or mcols()
#>
#>                name
#>   BFC32853 | GRCh37_to_GRCh38.chain
#>              Path
#>   BFC32853 /tmp/Rtmp6l0Pi8/SharedData/GRCh37_to_GRCh38.chain
dataNames(dh)
#> [1] "outfile.txt"                        "GRCh37_to_GRCh38.chain"
#> [3] "gencode.vM31.pc_transcripts.fa"     "gencode.vM31.pc_transcripts.fa.fai"
dataParams(dh)
#> [1] "input: Hello World!; outfile: outfile"
#> [2] "species: human; from: GRCh37; to: GRCh38"
#> [3] "species: mouse; version: M31"
#> [4] "species: mouse; version: M31"
dataNotes(dh)
#> [1] "echo txt test"                 "liftover human GRCh37 GRCh38"
#> [3] "gencode transcript mouse M31" "gencode transcript mouse M31"
dataTags(dh)
#> [1] "" "" "" ""
dataYml(dh)
#> [1] "/tmp/Rtmp6l0Pi8/SharedData/echo_hello_world.yml"
#> [2] "/tmp/Rtmp6l0Pi8/SharedData/ensembl_liftover_human_GRCh37_GRCh38.yml"
#> [3] "/tmp/Rtmp6l0Pi8/SharedData/rcp_mouse_M31.yml"
#> [4] "/tmp/Rtmp6l0Pi8/SharedData/rcp_mouse_M31.yml"
```

`ReUseData`, as the name suggests, commits to promoting the data reuse. Data can be prepared in standard input formats (`toList`), e.g., YAML and JSON, to be easily integrated in workflow methods that are locally or cloud-hosted.

```
(dd <- dataSearch(c("liftover", "GRCh38")))
#> dataHub with 1 records
#> cache path:  /home/qian/.cache/R/ReUseData
#> # dataUpdate() to update the local data cache
#> # dataSearch() to query a specific dataset
```

```
#> # Additional information can be retrieved using:
#> # dataNames(), dataParams(), dataNotes(), dataPaths(), dataTag() or mcols()
#>
#>                name
#>   BFC32853 | GRCh37_to_GRCh38.chain
#>              Path
#>   BFC32853 /tmp/Rtmp6l0Pi8/SharedData/GRCh37_to_GRCh38.chain
toList(dd)
#> $GRCh37_to_GRCh38.chain
#> [1] "/tmp/Rtmp6l0Pi8/SharedData/GRCh37_to_GRCh38.chain"
toList(dd, format = "yaml")
#> [1] "GRCh37_to_GRCh38.chain: /tmp/Rtmp6l0Pi8/SharedData/GRCh37_to_GRCh38.chain"
toList(dd, format = "json", file = "data.json") ## output to "file"
#> {
#>   "GRCh37_to_GRCh38.chain": "/tmp/Rtmp6l0Pi8/SharedData/GRCh37_to_GRCh38.chain"
#> }
```

Data can also be aggregated from different resources by tagging with specific software tools (`dataTags`).

```
dataTags(dh[1:2]) <- "#gatk"
dataSearch("#gatk")
#> dataHub with 2 records
#> cache path:  /home/qian/.cache/R/ReUseData
#> # dataUpdate() to update the local data cache
#> # dataSearch() to query a specific dataset
#> # Additional information can be retrieved using:
#> # dataNames(), dataParams(), dataNotes(), dataPaths(), dataTag() or mcols()
#>
#>                name
#>   BFC32852 | outfile.txt
#>   BFC32853 | GRCh37_to_GRCh38.chain
#>              Path
#>   BFC32852 /tmp/Rtmp6l0Pi8/SharedData/outfile.txt
#>   BFC32853 /tmp/Rtmp6l0Pi8/SharedData/GRCh37_to_GRCh38.chain
```

3.3 Data on the cloud

If the argument `cloud=TRUE` is enabled, `dataUpdate()` will also cache the pre-generated data sets (from evaluation of pre-built recipes) that are available on the `ReUseData` Google bucket and return those in the `dataHub` object that are fully searchable.

```
dh <- dataUpdate(dir = outdir, cloud = TRUE)
dataSearch(c("refGene"))
#> dataHub with 4 records
#> cache path:  /home/qian/.cache/R/ReUseData
#> # dataUpdate() to update the local data cache
#> # dataSearch() to query a specific dataset
#> # Additional information can be retrieved using:
#> # dataNames(), dataParams(), dataNotes(), dataPaths(), dataTag() or mcols()
#>
```

```
#>                name
#>   BFC32982 | refGene_hg38.sql
#>   BFC32983 | refGene_hg38.txt
#>   BFC32986 | refGene_mm39.sql
#>   BFC32987 | refGene_mm39.txt
#>              Path
#>   BFC32982 https://storage.googleapis.com/reusedata/ucsc_database/refGene_h...
#>   BFC32983 https://storage.googleapis.com/reusedata/ucsc_database/refGene_h...
#>   BFC32986 https://storage.googleapis.com/reusedata/ucsc_database/refGene_m...
#>   BFC32987 https://storage.googleapis.com/reusedata/ucsc_database/refGene_m...
```

If the data of interest already exist on the cloud, then `getCloudData` will directly download the data (and concomitant annotation files) to your computer. You can add it to the local caching system using `dataUpdate()` for later use.

```
dh <- dataSearch(c("refGene", "hg38"))
getCloudData(dh[1], outdir = outdir)
list.files(outdir, pattern = "refGene")
#> [1] "refGene_hg38.sql"               "UCSC_database_hg38_refGene.cwl"
#> [3] "UCSC_database_hg38_refGene.md5" "UCSC_database_hg38_refGene.sh"
#> [5] "UCSC_database_hg38_refGene.yml"
```

3.4 Know your data

Here we provide a function `meta_data()` to create a data frame that contains all information about the data sets in the specified file path (recursively), including the annotation file (`$yml` column), parameter values for the recipe (`$params` column), data file path (`$output` column), keywords for the data file (`notes` columns), and date and time information for data generation (`date` column).

Use `cleanup = TRUE` to cleanup any invalid or expired/older intermediate files.

```
mt <- meta_data(outdir, cleanup=TRUE)
head(mt)
#>                                                                   yml
#> 1                   /tmp/Rtmp6l0Pi8/SharedData/echo_hello_world.yml
#> 2 /tmp/Rtmp6l0Pi8/SharedData/ensembl_liftover_human_GRCh37_GRCh38.yml
#> 3                      /tmp/Rtmp6l0Pi8/SharedData/rcp_mouse_M31.yml
#> 4                      /tmp/Rtmp6l0Pi8/SharedData/rcp_mouse_M31.yml
#> 5          /tmp/Rtmp6l0Pi8/SharedData/UCSC_database_hg38_refGene.yml
#>                                  params
#> 1    input: Hello World!; outfile: outfile
#> 2 species: human; from: GRCh37; to: GRCh38
#> 3              species: mouse; version: M31
#> 4              species: mouse; version: M31
#> 5              build: hg38; dbname: refGene
#>                                                              output
#> 1                        /tmp/Rtmp6l0Pi8/SharedData/outfile.txt
#> 2              /tmp/Rtmp6l0Pi8/SharedData/GRCh37_to_GRCh38.chain
#> 3     /tmp/Rtmp6l0Pi8/SharedData/gencode.vM31.pc_transcripts.fa
#> 4 /tmp/Rtmp6l0Pi8/SharedData/gencode.vM31.pc_transcripts.fa.fai
```

```
#> 5                  /tmp/Rtmp6l0Pi8/SharedData/refGene_hg38.sql
#>                                 notes                date
#> 1                       echo txt test 2023-11-10 21:05:49
#> 2          liftover human GRCh37 GRCh38 2023-11-10 21:06:44
#> 3          gencode transcript mouse M31 2023-11-10 21:06:39
#> 4          gencode transcript mouse M31 2023-11-10 21:06:39
#> 5 ucsc annotation database hg38 refGene          2022-12-21
```

4 SessionInfo

```
sessionInfo()
#> R version 4.2.2 Patched (2022-11-10 r83330)
#> Platform: x86_64-pc-linux-gnu (64-bit)
#> Running under: Ubuntu 20.04.3 LTS
#>
#> Matrix products: default
#> BLAS:   /usr/lib/x86_64-linux-gnu/blas/libblas.so.3.9.0
#> LAPACK: /usr/lib/x86_64-linux-gnu/lapack/liblapack.so.3.9.0
#>
#> locale:
#>  [1] LC_CTYPE=en_US.UTF-8       LC_NUMERIC=C
#>  [3] LC_TIME=en_US.UTF-8        LC_COLLATE=en_US.UTF-8
#>  [5] LC_MONETARY=en_US.UTF-8    LC_MESSAGES=en_US.UTF-8
#>  [7] LC_PAPER=en_US.UTF-8       LC_NAME=C
#>  [9] LC_ADDRESS=C               LC_TELEPHONE=C
#> [11] LC_MEASUREMENT=en_US.UTF-8 LC_IDENTIFICATION=C
#>
#> attached base packages:
#> [1] stats4    stats     graphics  grDevices utils     datasets  methods
#> [8] base
#>
#> other attached packages:
#> [1] Rcwl_1.15.6         S4Vectors_0.36.2    BiocGenerics_0.44.0
#> [4] yaml_2.3.7          ReUseData_0.99.38   BiocStyle_2.26.0
#>
#> loaded via a namespace (and not attached):
#>  [1] httr_1.4.5            bit64_4.0.5           jsonlite_1.8.4
#>  [4] here_1.0.1            R.utils_2.12.2        shiny_1.7.4
#>  [7] BiocManager_1.30.20   BiocFileCache_2.6.1   base64url_1.4
#> [10] blob_1.2.3            remotes_2.4.2         progress_1.2.2
#> [13] lattice_0.20-45       pillar_1.8.1          RSQLite_2.3.0
#> [16] backports_1.4.1       reticulate_1.28       glue_1.6.2
#> [19] digest_0.6.31         RColorBrewer_1.1-3    promises_1.2.0.1
#> [22] checkmate_2.1.0       Matrix_1.4-0          htmltools_0.5.4
#> [25] httpuv_1.6.9          R.oo_1.25.0           pkgconfig_2.0.3
#> [28] dir.expiry_1.6.0      bookdown_0.33         DiagrammeR_1.0.9
#> [31] purrr_1.0.1           RcwlPipelines_1.14.0  xtable_1.8-4
#> [34] processx_3.8.0        brew_1.0-8            later_1.3.0
#> [37] BiocParallel_1.32.5   git2r_0.31.0          tibble_3.2.0
```

```
#> [40] generics_0.1.3       ellipsis_0.3.2       cachem_1.0.7
#> [43] withr_2.5.0          cli_3.6.0            magrittr_2.0.3
#> [46] crayon_1.5.2         mime_0.12            memoise_2.0.1
#> [49] evaluate_0.20        ps_1.7.2             R.methodsS3_1.8.2
#> [52] fansi_1.0.4          pkgbuild_1.4.0       tools_4.2.2
#> [55] data.table_1.14.8    prettyunits_1.1.1    hms_1.1.2
#> [58] lifecycle_1.0.3      basilisk.utils_1.10.0 callr_3.7.3
#> [61] compiler_4.2.2       tinytex_0.44         rlang_1.1.0
#> [64] grid_4.2.2           rappdirs_0.3.3       htmlwidgets_1.6.1
#> [67] visNetwork_2.1.2     basilisk_1.10.2      rmarkdown_2.20
#> [70] codetools_0.2-18     DBI_1.1.3            curl_5.0.0
#> [73] R6_2.5.1             knitr_1.42           dplyr_1.1.0
#> [76] fastmap_1.1.1        bit_4.0.5            utf8_1.2.3
#> [79] filelock_1.0.2       rprojroot_2.0.3      desc_1.4.2
#> [82] stringi_1.7.12       parallel_4.2.2       Rcpp_1.0.10
#> [85] png_0.1-8            vctrs_0.5.2          dbplyr_2.3.2
#> [88] batchtools_0.9.16    tidyselect_1.2.0     xfun_0.37
```
